# Photofungizides Based on Curcumin and Derivates Thereof against *Candida albicans* and *Aspergillus niger*

**DOI:** 10.3390/antibiotics10111315

**Published:** 2021-10-28

**Authors:** Barbara Schamberger, Kristjan Plaetzer

**Affiliations:** 1 Laboratory of Photodynamic Inactivation of Microorganisms, Department of Biosciences, Paris Lodron University of Salzburg, 5020 Salzburg, Austria; barbara.schamberger@plus.ac.at; 2 Morphophysics Group, Department of Chemistry and Physics of Materials, Paris Lodron University of Salzburg, 5020 Salzburg, Austria

**Keywords:** photodynamic inactivation, photosensitizer, antifungal, curcumin, *Candida albicans*, *Aspergillus niger*

## Abstract

Fungal infections in humans, contamination of food and structural damage to buildings by fungi are associated with high costs for the general public. In addition, the increase in antifungal resistance towards conventional treatment raises the demand for new fungicidal methods. Here, we present the antifungal use of Photodynamic Inactivation (PDI) based on the natural photosensitizer curcumin and a water-soluble positively charged derivative thereof (SA-CUR 12a) against two different model organisms; *Candida albicans* grown in a liquid culture and photo treated with a 435 nm LED light followed by counting of the colony-forming units and photoinactivation of tissue-like hyphal spheres of *Aspergillus niger* (diameter ~5 mm) with subsequent monitoring of colony growth. Curcumin (50 µM, no incubation period, i.p.) supplemented with 10% or 0.5% DMSO as well as SA-CUR 12a (50 µM no i.p or 5 min i.p.) triggered a photoantifungal effect of >4 log units towards *C. albicans*. At 100 µM, SA-CUR 12a (0 min or 5 min i.p.) achieved a reduction of >6 log units. Colonies of *A. niger* shrunk significantly during PDI treatment. Photoinactivation with 50 µM or 100 µM curcumin (+0.5% DMSO) resulted in complete growth inhibition. PDI using 20, 50 or 100 µM SA-CUR 12a (with or without 10% DMSO) also showed a significant reduction in colony area compared to the control after 48 h, although less pronounced compared to curcumin. In summary, PDI using curcumin or SA-CUR 12a against *C. albicans* or *A. niger* is a promising alternative to currently used fungicides, with the advantage of being very unlikely to induce resistance.

## 1. Introduction

From the kingdom of fungi, about 600 species are known to be human pathogens, that can be potentially harmful to immunocompromised people (e.g., *Candida* spp. or *Aspergillus* spp.) [1]. As the number of immuno-deficient people increases due to the rising number of patients undergoing cancer therapy, organ recipients or people treated for human immunodeficiency virus, the risk of contracting a fungal infection also increases [2,3,4]. In addition, an increase in antimicrobial resistance is observed in recent years [5]. Fungi in the form of mold in buildings cause high costs as well as an increased health risk for the occupants [6]. Therefore, new antifungal agents are needed to counter the increasing number of fungal infections and the increasing antifungal resistance against conventional agents [5].

Photodynamic Inactivation (PDI), also known as Antimicrobial Photodynamic Therapy, is a method that was shown to be highly effective against bacteria [7,8,9,10,11,12]. PDI uses a photoactive substance, or photosensitizer (PS), which, together with oxygen and excitation by light of an appropriate wavelength, generates reactive oxygen species (ROS) [13]. Unlike antibiotics, PDI is not linked to a specific target in the organism, which is why the development of resistance to PDI is unlikely but must still be considered [14,15]. Two different model organisms were selected for the investigation of the potential antifungal effect of PDI: *Candida albicans* (*C. albicans*) and *Aspergillus niger* (*A. niger*).

*Candida albicans* is a yeast-like fungus that is harmless in most cases and is found in the oral cavity of over 75% of the population. Immunocompromised people can suffer from candidiasis in the oral cavity and up to 75% of women report having had a vaginal infection with *Candida* spp. at least once in their lifetime. Although these very common superficial infections are not life-threatening, *Candida* spp. can also lead to systemic infection, where the mortality rate is very high [1]. Antifungal resistance is an increasing problem with *Candida*. About 7% of all *Candida* blood samples tested at the Centers for Disease Control (CDC) are resistant to the antifungal drug fluconazole [16]. Very special concern is rising over *Candida auris*, which is still rare but a growing threat. Resistance rates for *C. auris* are much higher than for other *Candida* species, with about 90% of U.S. *C. auris* isolates being resistant to fluconazole, and one-third resistant to amphotericin B [17].

*A. niger* is a filamentous fungus that can develop conidiophores with black spores [18] and is among other fungal species causing mold growth. Dampness and mold growth in a high humidity environment caused by leaks, inadequate ventilation or extreme natural events (e.g., floods) lead to an increase in respiratory diseases such as asthma and therefore have a major impact on health care costs [6]. In immunocompromised patients *Aspergillus* spp. can lead to *Aspergillus* infections, which can even be life-threatening in case of severe septic infections [19]. The shift to energy-efficient housing using air-tight constructions also leads to more favorable conditions for mold growth [20]. It is estimated that people spend about 90% of their time indoors, so the impact of poor housing conditions, such as those caused by mold, on occupants’ health is very high [21]. The most effective step towards mold-free homes is to eliminate the source of moisture in a building and remove materials that are already contaminated. Additionally, biocides such as chlorine bleach might be used, but unfortunately, biocides are also harmful to humans [22]. In the CDC report “Antibiotic resistance threats in the United States 2019”, *Aspergillus fumigatus* is placed on the watch list because of the rise in azole-resistant infections [23].

In PDI a wide spectrum of PSs is known that have already shown high efficiency against bacteria or fungi. However, the use of natural substances such as curcumin, which is classified as a food additive (E100), appears to be a good choice for mild, eco-friendly and biocompatible photoangifungal treatment, as it forms ROS in an appropriate environment [24,25]. Curcumin was shown to be *effective* against bacteria, e.g., *Staphylococcus aureus* [26] or fungi, e.g., *A. niger* [27] or *C. albicans* [28]. Due to its low solubility in water, curcumin is often dissolved in a solubility enhancer, e.g., 10% dimethyl sulfoxide (DMSO) [26,28] or propylene glycol [27] and also used as formulations with solubility enhancers [24]. Alternatively, water-soluble curcumin derivates can be used [29,30,31].

In this study, we introduce SA-CUR 12a, a new curcumin-based cationic PS for anti-fungal treatment and compare it to natural curcumin, to demonstrate the potential of PDI as an alternative to conventional fungicides. The impact of DMSO on curcumin-based antifungal PDI is determined by using two different concentrations of this solubility enhancer (0.5% and 10%). In the study, two model systems with fundamental distinct growth behavior are used: the yeast-like fungus *C. albicans*, which is of clinical relevance, and the filamentous *A. niger* having environmental importance.

## 2. Results

### 2.1. PDI against Candida albicans with Curcumin or SA-CUR 12a

**Figure 1:**
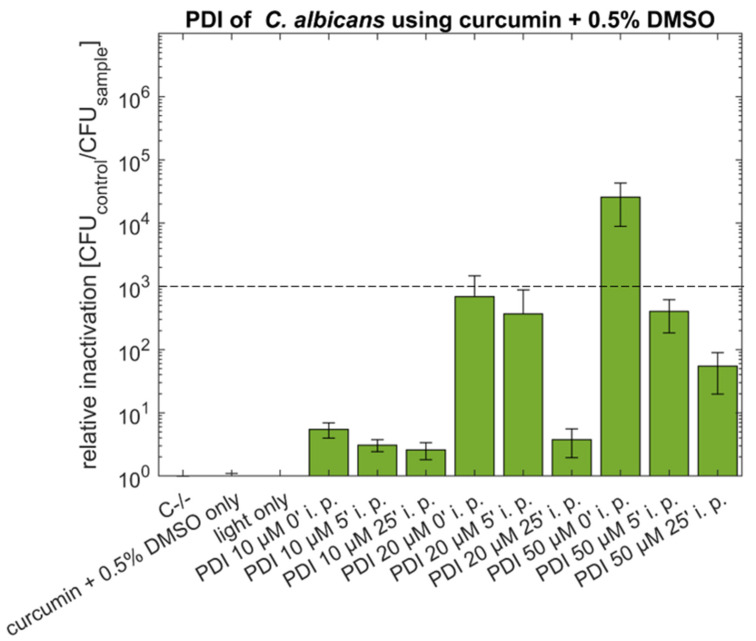
Phototoxicity of curcumin (10 µM, 20 µM or 50 µM) + 0.5% DMSO against *C. albicans* using incubation periods (i.p.) of 0, 5 or 25 min. Illumination was performed by using an LED array (15.8 J cm^−2^/435 nm). The plot shows the mean and the standard deviation of the relative inactivation according to C−/− (*n* (number of biological replicas) = 3). The dotted line indicates a 3 log reduction.

To determine the effect of the DMSO concentration, the natural PS curcumin with 0.5% or 10% DMSO was used against the pathogenic yeast *C. albicans* in the initial experiments. This solubility enhancer does not have a toxic effect: as shown in Figure 1, the mean CFU of the individual control samples (C−/−, light only, PS only) are in the same order of magnitude (CFU ranging from 1.5–5 × 10^6^).

Curcumin dissolved with 0.5% DMSO showed an antimicrobial effect (reduction of 3 log) of 2.6 × 10^4^ only in the concentration 50 µM without incubation period (i. p.) before illumination. Using a five-minute incubation with the PS before illumination resulted in a decrease in relative inactivation (rel. inactivation 4.0 × 10^2^), which was even more pronounced (rel. inactivation 5.5 × 10^1^), if the incubation period was extended to 25 min.

**Figure 2:**
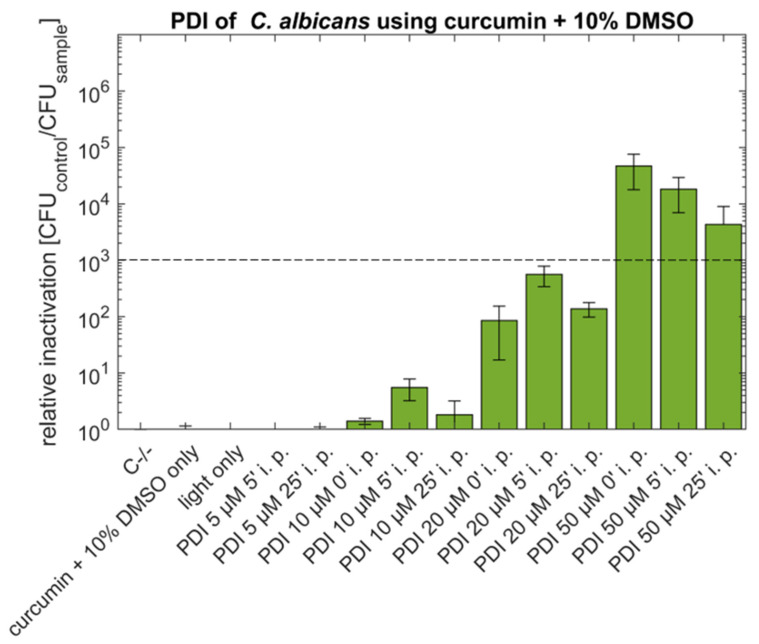
Phototoxicity of curcumin (5 µM, 10 µM, 20 µM or 50 µM) + 10% DMSO against *C. albicans* using incubation periods (i.p.) of 0, 5 or 25 min. Illumination was performed by using an LED array (15.8 J cm^−2^/435 nm). The plot shows the mean and the standard deviation of the relative inactivation according to C−/− (*n* = 3). The dotted line indicates a 3 log reduction.

After increasing the concentration of supplemented DMSO to 10% (Figure 2) the antimycotic effect of 50 µM curcumin and no incubation before illumination was in the same order of magnitude (rel. inactivation 4.7 × 10^4^) as for the lower concentration of DMSO. The effect of the treatments with 50 µM curcumin and incubation times of 5 min (rel. inactivation 1.8 × 10^4^) or 25 min (rel. inactivation 4.3 × 10^3^) lead to lower efficiency, but the effect still remains above 3 log. Lower concentrations (5, 10 and 20 µM) of curcumin with 10% DMSO induced no antimicrobial effect.

**Figure 3:**
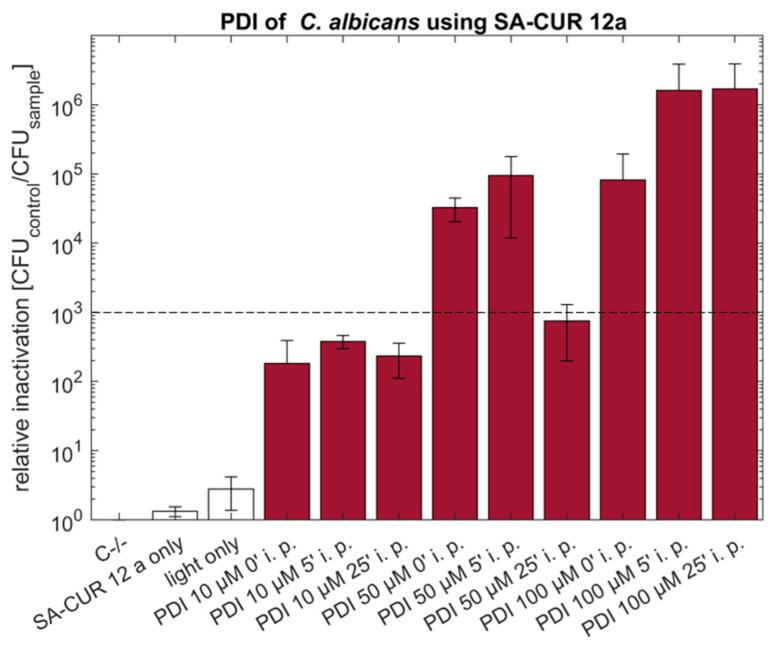
Phototoxicity of SA-CUR 12a (10 µM, 50 µM or 100 µM) against *C. albicans* using incubation periods (i.p.) of 0, 5 or 25 min. Illumination was performed by using an LED array (15.8 J cm^−2^/435 nm). The plot shows the mean and the standard deviation of the relative inactivation according to C−/− (*n* = 3). The dotted line indicates a 3 log reduction.

Photodynamic Inactivation based on the water-soluble cationic SA-CUR 12a at concentrations of 50 µM without incubation induced an antimycotic effect (3.3 × 10^4^) comparable to 50 µM curcumin without incubation. Increasing the incubation time (i.p. 5 min) slightly increased the effect to 9.5 × 10^4^. A 25 min incubation showed no improvement but led to a drop in efficiency below the 3 log criteria to a relative inactivation of 7.5 × 10^2^. Increasing the concentration of SA-CUR 12a to 100 µM and incubation for 5 or 25 min prior to illumination resulted in a pronounced decimation of viable *C. albicans* of at least 1.6 × 10^6^, which was at the detection limit (Figure 3).

### 2.2. PDI against Aspergillus niger with Curcumin or SA-CUR 12a

**Figure 4:**
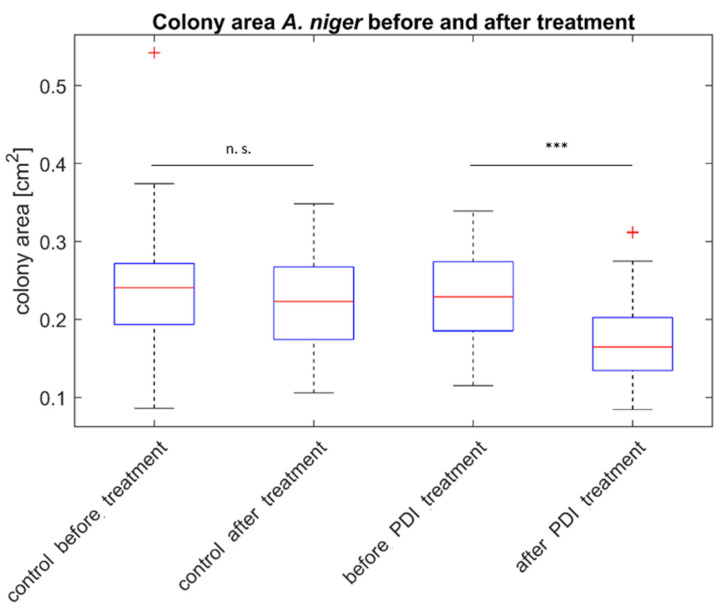
Colony area of mycelial patches of *A. niger* before and after treatment. The plot shows the median and the standard deviation of the colony area. Statistical significance of the area before and after treatment is indicated with no significance (n. s.), *p* < 0.001 (***). *n*_control_ = 33, *n*_treatment_ = 60.

To determine whether PDI treatment leads to a loss of the cell mass immediately after illumination the colony areas of the *A. niger* mycelial patches were measured before and directly after illumination. Figure 4 displays the diameters of mycelial patches (i.e., colony areas) of *A. niger* photo treated with PS as well as the control groups (C−/−, light only, PS only) before and after illumination. A significant decrease in patch size is seen post-treatment. The list of samples included in each group can be found in the Appendix A.

**Figure 5:**
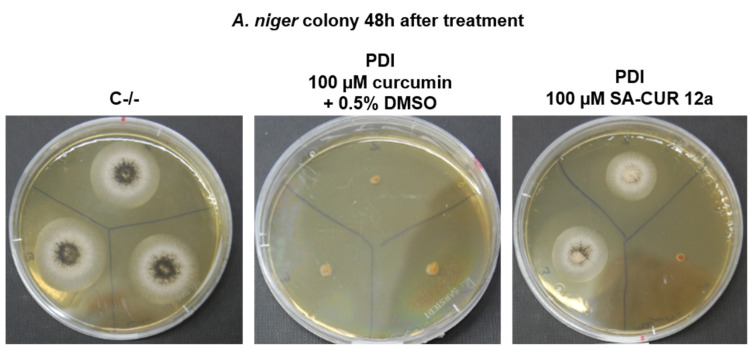
*A. niger* 48 h after PDI treatment. Left: control sample (C−/−), Middle: PDI treated samples with 100 µM curcumin + 0.5% DMSO, Right: PDI treated samples with 100 µM SA-CUR 12a.

After incubating the samples at growth conditions for 48 h post-treatment it can be seen that the control sample developed black spores, while the development slows down in the samples treated with 100 µM SA-CUR12a and after 48 h there are fewer or no spores (Figure 5). In colonies treated with 100 µM curcumin + 0.5% DMSO, growth was completely inhibited and no new mycelium was formed.

**Figure 6:**
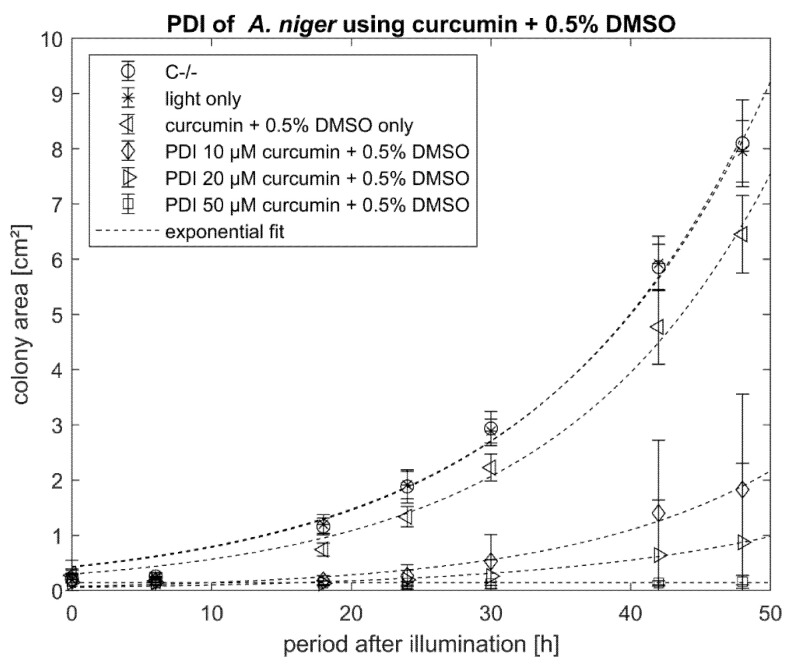
Phototoxicity of curcumin + 0.5% DMSO (10 µM, 20 µM, 50 µM) against *A. niger*. The incubation period before illumination was 20 min. Illumination was performed with 15.8 J cm^−2^/435 nm. Plotted is the mean and standard deviation (*n* = 9). The dotted line indicates an exponential fit.

Looking at the growth of the colonies after treatment over time, it can be seen that the control samples C−/− and light only show a similar growth behavior: the PS control (curcumin + 0.5% DMSO) without illumination already reduced growth rate. Using at least 50 µM of curcumin with 0.5% DMSO, the growth was completely suppressed. Lower concentrations of curcumin (10 µM, 20 µM + 0.5% DMSO) still resulted in a significant difference in the grown area after 48 h (Figure 6).

**Figure 7:**
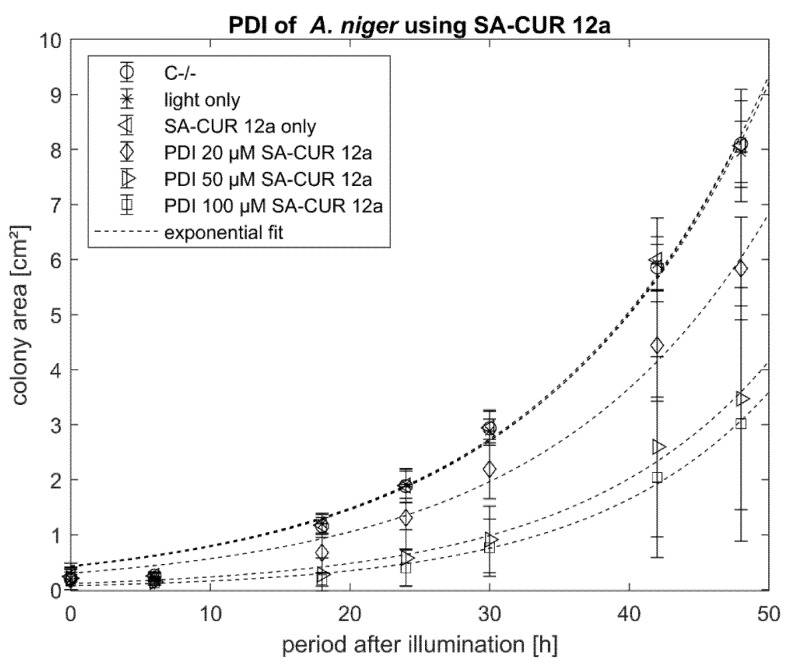
Phototoxicity of SA-CUR 12a (20 µM, 50 µM or 100 µM) against *A. niger*. The incubation period before illumination was 20 min. Illumination was performed with 15.8 J cm^−2^/435 nm. Plotted is the mean and standard deviation (*n* = 9). The dotted lines indicate an exponential fit.

The cationic SA-CUR 12a did not induce any dark toxicity (Figure 7). The highest phototoxicity of SA-CUR 12a was observed at a concentration of 100 µM with a mean colony area after 48 h of 3.0 mm^2^. At lower concentrations, the photoantimycotic effect was less pronounced with decreasing concentration of SA-CUR 12a. 

**Figure 8:**
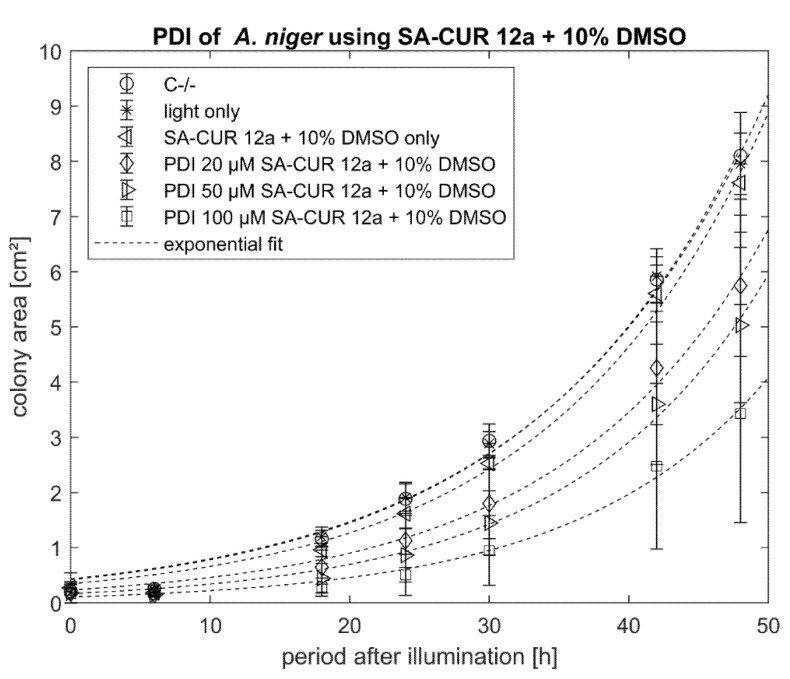
Phototoxicity of SA-CUR 12a + 10% DMSO (20 µM, 50 µM, 100 µM) against *A. niger*. The incubation period before illumination was 20 min. Illumination was performed with 15.8 J cm^−2^/435 nm. Plotted is the mean and standard deviation (*n* = 9). The dotted lines indicate an exponential fit.

To examine whether the phototoxicity of SA-CUR 12a can be increased with DMSO, experiments with 10% of this permeability enhancer were performed. For SA-CUR 12a with 10% DMSO, the highest antimicrobial effect of SA-CUR 12a was achieved with 100 µM (mean colony area after 48 h of 3.4 mm^2^, see Figure 8).

**Figure 9:**
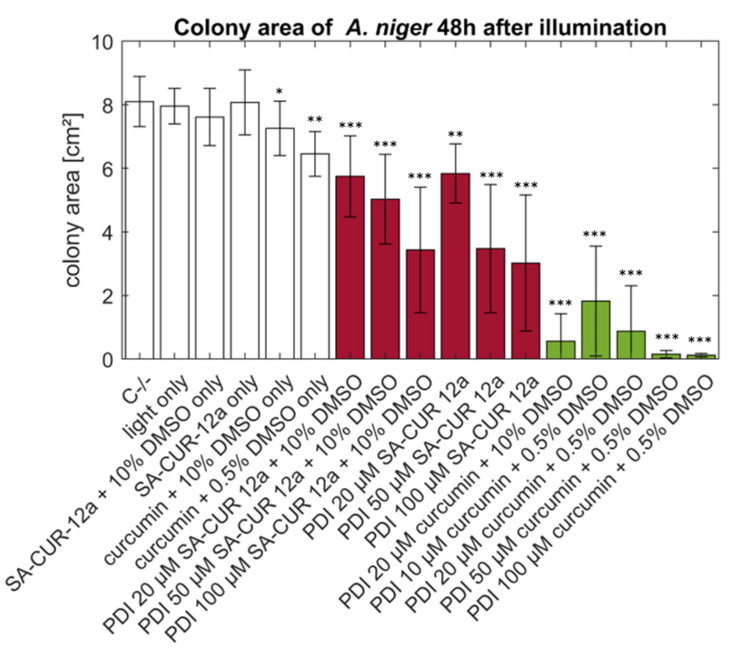
Summary of phototoxicity of SA-CUR-12a (red) and curcumin (green) in different concentrations and different supplementation of DMSO against *A. niger*. The incubation period before illumination was 20 min. The plotted colony area was determined after 48 h post-treatment. Illumination was performed with 15.8 J cm^−2^/435 nm. Plotted is the mean and standard deviation (*n* = 9). Statistical significance between individual treatments and C−/− was indicated with *p* < 0.05 (*), *p* < 0.005 (**), *p* < 0.001 (***).

Figure 9 summarizes the colony areas of *A. niger* 48 h post-treatment. Curcumin in combination with DMSO induces significant (*p* < 0.05) dark toxicity. The individual treatments do all show a significant decrease in the colony area when compared to C−/−. The lowest effect is seen with 20 µM SA-CUR 12a and the highest with curcumin with 10% DMSO at the concentrations 50 µM and 100 µM, inducing complete growth inhibition.

## 3. Discussion

Fungal infections in humans as well as fungal contamination of buildings or in food causes high costs for society [6,32]. In the medical sector, there are only a small number of substances approved for the treatment of fungal infection, with the drawback of having the potential to develop an antimicrobial resistance [5]. Indeed, the resistance of *Candida* against fluconazole is increasing alarmingly: Since the discovery of multidrug-resistant *Candida* spp. *C. auris* in Japan, outbreaks were detected in hospitals in several countries around the globe [33]. The majority (>90%) of *C. auris* isolates are resistant to fluconazole, an anti-fungal drug widely used in hospitals [17]. This is of particular concern as the availability of approved antifungal treatments is very limited [5]. 

In the case of fungal infestation, e.g., mold in buildings, some of the agents used are harmful to the inhabitants [22]. Conventional chemical disinfectants to combat fungi often contain bleach, which is a highly reactive substance, or ethanol, which is easily flammable. While bleach is effective against fungi, it also poses a health risk to the user as it can irritate the skin and respiratory tract. Therefore the use of these two substances should therefore be restricted to ventilated rooms [34]. Due to the aforementioned disadvantages of conventional antifungal agents, this study focuses on the use of PDI against two fungal species with different growth behavior.

PDI using curcumin or curcumin formulations was previously proven to be highly efficient against bacteria [24,26]. In addition, PDI also has the potential to be used against other microbial targets, such as viruses, parasites or even fungi [8]. Due to their relevance in many aspects, more and more research is looking into the potential of PDI to combat fungi [28,35,36,37,38]. Therefore, we have shown here that PDI using curcumin and a water-soluble derivate of curcumin (SA-CUR 12a) shows potential as an alternative to conventional treatment.

To demonstrate the potential of the photoantifungal approach for clinical application, *C. albicans* was used as a model organism. Although 50 µM curcumin with 10% DMSO (i.p. 0′) showed an antimicrobial effect of 4.7 log steps, the efficiency is lower when compared to the phototoxicity reported by Dovigo and coworkers [28]. In this study, the authors showed complete eradication (~6 log) of *C. albicans* using lower curcumin concentrations (20 µM curcumin + 10% DMSO, i.p. 20 min) and comparable light conditions (different light fluences from 5.28–26.4 J cm^−2^, compared to 15.8 J cm^−2^ in the current study). For comparison, in our study, no antimicrobial effect was observed at 20 µM curcumin + 10% DMSO (i.p. 0, 5 or 25 min, see Figure 2). Interestingly, shorter incubation periods result in more pronounced photokilling of fungi, indicative of the limited stability of curcumin in aqueous solution influencing the photoantifungal effect.

By reducing the DMSO content to a final concentration of 0.5% (50 µM curcumin + 0.5% DMSO, i.p. 0′) the relative inactivation was in the same order of magnitude (>4 log). Again, the phototoxic effect decreased with increasing incubation period before illumination. Reducing the concentration of DMSO in PDI using curcumin could prove beneficial, as DMSO has a broad spectrum of effects (e.g., anti-inflammatory or ROS scavenger) which could counteract photo treatment [39,40]. Therefore, the influence of DMSO needs to be considered when applying the method in medicinal applications. In particular, DMSO could scavenge ROS generated by PDI.

When using the water-soluble curcumin derivate SA-CUR 12a as a photosensitizer, the supplementation of DMSO becomes obsolete. The photofungal effect against *C. albicans* (50 µM SA-CUR 12a, i.p. 0 or 5 min) was in the same order of magnitude (<4 log) as for curcumin. When the concentration of SA-CUR 12a was further increased to 100 µM (i.p. 5 or 25 min), maximal relative inactivation (>6 log steps) was induced.

Given the high photokilling efficiency of curcumin and SA-CUR 12a, PDI using these two substances is a promising alternative for the treatment of *C. albicans* infections. The use of curcumin in PDI against oral candidiasis has already been tested in immunosuppressed mice showing promising results (4 log reduction with treatment using 80 µM curcumin + 10% DMSO, 37.5 J cm^−2^) [41]. Additionally, first tests were performed on the clinical application of PDI for the treatment of oral candidiasis [37] using methylene blue as a photoactive compound. This approach induced a reduction of 1.99 log CFU of *Candida* spp. (mainly *C. tropicalis* and *C. glabrata*) after treatment of oral candidiasis in mechanically ventilated patients.

To provide insight into the potential of antifungal PDI in an environmental application, the model organism *A. niger* was used. This fungal species is also very interesting for comparison to *Candida*, as the experimental approach for determination of the photoantimicrobial effect is very different: viable spheres of *A. niger* with a diameter of 5 mm, thus representing tissue-like structures with the need for the diffusion of the photoactive compound into the mycelial patches, where exposed to curcumin or SA-CUR 12a at different concentration for 20 min and subsequently illuminated with light (15.8 J cm^−2^). The growth of colonies was monitored for 48 h. It was found that the treatment resulted in a significant decrease in colony size after illumination compared to the control samples. Fungal patches showing no increase in size were considered dead.

SA-CUR 12a was tested in three different concentrations (20, 50, 100 µM). To assess the influence of DMSO on the penetration of the PD into the spheres, experiments with or without 10% DMSO were performed. All combinations reduced the size of mycelial patches of *A. niger* after 48 h but did not completely inhibit the growth of the fungus. On the contrary, using curcumin as a PS increased the antifungal effect with a total growth inhibition using concentrations of 50 µM or 100 µM, both supplemented with 0.5% DMSO. Curcumin was found to show significant dark toxicity at 20 µM + 10% DMSO (*p* < 0.05) or 100 µM + 0.5% DMSO (*p* < 0.005).

Figure 5 shows that in untreated controls the typical *Aspergillus* black conidia are formed after incubation on agar plates after 48 h. In contrast, if patches are treated with 100 µM SA-CUR 12a the growth rate is slowed down and fewer or no conidia are formed. If 100 µM curcumin + 0.5% DMSO is used as a photoactive agent, there is no growth of mycelia and no conidia formation.

PDI has already been used to treat *A. niger* in previous studies; the PS used were synthetic substances (charged corrosoles and conjugated polymers) which, in contrast to the viable colonies presented here, were used to treat conidia [35,36]. Growth inhibition was observed when at least 5 µM PTP (polythiophene-porphyrin) and the illumination of 50 J cm^−2^ were used. The viability of *A. niger* was reduced to 15% with 20 µM PTP [36]. PDI with two different cationic corrosoles at much higher concentrations when compared to our study (1 mM PS incubated for three days with constant illumination of 9 mW cm^−2^) resulted in a complete growth arrest [35]. The use of curcumin could however prove to work as a very mild, cheap, biocompatible and eco-friendly alternative to synthetic photosensitizers.

Taking the presented work and the work from literature together, PDI should be considered as a novel strategy against fungal contamination in an environmental context but also in the food industry, as curcumin is approved as a food additive E100.

## 4. Conclusions

In conclusion, the results of using the novel PS SACUR-12a and natural curcumin in PDI treatment show multiple applications in clinical and industrial settings. As *C. albicans* and *A. niger* are culprits for several health problems in humans and the current treatment is expensive and leads to problems such as antimicrobial resistance or pulmonary diseases, the use of PDI seems to be a promising alternative.

## 5. Materials and Methods

### 5.1. Preparation of PS Solutions

**Figure 10:**
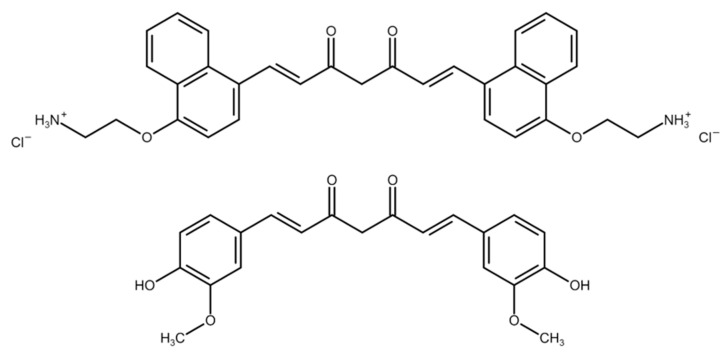
Chemical structures of SA-CUR 12 a (**top**) and curcumin (**bottom**).

The stock solution (100 mM) of curcumin (Carl Roth, Karlsruhe, >90.6%) was prepared by dissolving in DMSO (Fluka Analytical, Buchs, Switzerland). SACUR-12a [42] was kindly provided by Dr. Andreas Spaeth (Institute of Organic Chemistry, University of Regensburg, Regensburg, Germany). A stock solution (5 mM) of SACUR-12a was prepared in ddH_2_O. Both stock solutions were stored at −20 °C in the dark until use. The chemical structures of curcumin and SA-CUR 12a [42] are shown in Figure 10. 

### 5.2. Culture of C. albicans

*Candida albicans* (Mya 273, ATCC) was grown in 20 mL Sabouraud Broth (SB) media containing 32 g L^−1^ peptone from casein tryptic digested (Carl Roth), 20 g L^−1^ yeast extract (AppliChem, Darmstadt, Germany), 5 g L^−1^ sodium chloride (VWR, PA, USA), and 5 mM sodium hydroxide (Fluka Analytical, Buchs, Switzerland) overnight at 37 °C under constant agitation (200 rpm).

### 5.3. PDI against C. albicans

The overnight culture was diluted with SB media to absorption of 0.05 AU at 600 nm (Infinite M200, Tecan, Switzerland) and incubated for 4 h at 37 °C and 200 rpm to a final concentration of about 0.3 AU at 600 nm (corresponding to about 6.5 log CFU per 2 mL culture). For each treatment, 2 mL of the cultures were centrifuged at 830 rcf for 3 min (Centrifuge 5417R, Eppendorf, Hamburg, Germany). The supernatant was removed and the pellet was resuspended in 1 mL sterile filtered DPBS (Dulbecco’s Phosphate Buffered Saline, Lonza Group, Verviers, Belgium) containing the different concentrations of the photosensitizer (10 µM, 50 µM or 100 µM SACUR-12a; 5 µM, 10 µM, 20 µM or 50 µM curcumin with 0.5% or 10% DMSO). Three controls were used for each experiment: C−/− (without PS and illumination), PS only (with PS at the highest concentration used in the experiment and no light), and light only (no PS, but with illumination). The samples were then incubated in the dark for different periods of time (0, 5 or 25 min). All control samples were incubated for 25 min. Subsequently, the samples and the light control were illuminated from below with a self-made LED array (435 nm, 15.8 J cm^−2^) under constant shaking (400 rpm) for 1 h. The C−/− and PS-only control were incubated under constant shaking (400 rpm) in the dark for 1 h. The samples were serially diluted in DPBS and plated on SB plates containing 1.5% Agar-Agar Kobe I (Carl Roth) and incubated for one day at 37 °C followed by counting the colony-forming unit (CFU). All experiments were repeated three times.

### 5.4. Culture of A. niger

Conidida from clinical isolates of *Aspergillus niger*, kindly provided by PD Dr. Tim Maisch (University Regensburg), were harvested using a sterile inoculation loop. The conidia were transferred to 20 mL SB media containing 0.2 mM Tween-20 (Carl Roth GmbH + CoKg) and incubated for two days at 37 °C in a 50 mL Greiner tube with constant shaking (200 rpm) (method adapted from [43]). A total of 1 mL of the liquid culture was used to inoculate 30 mL of fresh SB media. This subculture was incubated for two days at 37 °C in a 50 mL Greiner tube with constant shaking (200 rpm) before being used for PDI.

### 5.5. PDI against A. niger

Single colonies (sphere diameter: 5 mm) of *A. niger* were transferred to 500 µL of treatment solutions containing different concentrations of PS diluted in DBPS. Three controls were used for each experiment: C−/− (without a PS and illumination), PS control (with PS in the highest concentration used in the experiment and no light) and light control (illumination with the samples but without a PS). The samples were then incubated for 20 min in the dark at room temperature with constant shaking (400 rpm). Afterward, the samples, except for C−/− and PS control, were illuminated with an LED array (435 nm, 15.8 J cm^−2^) from below under constant shaking (400 rpm). The C−/− and the PS control were incubated under constant shaking in the dark for one hour using the same shaker as the samples. The colonies were then transferred to an SB agar plate containing 1.5% Agar-Agar Kobe I (for microbiology, Carl Roth GmbH + CoKg) and incubated for three days at 37 °C (adapted from [43]).

Growth inhibition was investigated by imaging the cultures at different time points and measuring the colony area using the image processing and analyzing software ImageJ 1.48v (National Institute of Health, Bethesda, MD, USA [44]). Each experiment was reproduced nine times.

### 5.6. Data Analysis

Unless otherwise stated, data represent the mean with the corresponding error bars of standard deviation of the relative inactivation (*C. albicans*, *n* = 3) or the colony area (*A. niger*, *n* = 9). The relative inactivation was calculated by dividing the CFU of the corresponding C−/− by the CFU of the individual treatments. In the case of complete inactivation (CFU = 0) the CFU of the C−/− was divided by one [29]. The dashed line in the *C. albicans* plots represents a 3 log reduction, which marks the minimum reduction for an antimicrobial effect. Data analysis was performed using Excel (v2016, Microsoft, Redmond, WA, USA) and Matlab R2019a (MathWorks, Natick, MA, USA). Statistical analysis was performed using RStudio version 1.4 (Bosten, MA, USA) using the Mann–Whitney U test. Statistical significance was indicated with *p* < 0.05 (*), *p* < 0.005 (**), *p* < 0.001 (***).

## Data Availability

The data presented in this study are available within this article.

## Abbreviations

*A. niger*	*Aspergillus niger*
*C. albicans*	*Candida albicans*
CFU	Colony forming unit(s)
DPBS	Dulbecco’s Phosphate Buffered Saline
i.p.	incubation period
PDI	Photodynamic Inactivation
PS	Photosensitiser
ROS	Reactive oxygen species
SB	Sabouraud Broth

## Appendix A

Values represented in Figure 4 are including the following samples:

**Control before and after Illumination**	**Treatment before and after Illumination**
C−/− [*n* = 6]	PDI 10 µM curcumin + 0.5% DMSO [*n* = 9]
Light only [*n* = 6]	PDI 20 µM curcumin + 0.5% DMSO [*n* = 6]
Curcumin + 0.5% DMSO only [*n* = 9]	PDI 50 µM curcumin + 0.5% DMSO [*n* = 9]
SA-CUR 12a only [*n* = 6]	PDI 20 µM SA-CUR 12a [*n* = 6]
SA-CUR 12a + 10% DMSO only [*n* = 6]	PDI 50 µM SA-CUR 12a [*n* = 6]
	PDI 100 µM SA-CUR 12a [*n* = 6]
	PDI 20 µM SA-CUR 12a + 10% DMSO [*n* = 6]
	PDI 50 µM SA-CUR 12a + 10% DMSO [*n* = 6]
	PDI 100 µM SA-CUR 12a + 10% DMSO [*n* = 6]
